# Supplementary material for: Deep learning connects DNA traces to transcription to reveal predictive features beyond enhancer–promoter contact
Source: Nat Commun. 2021 Jun 8;12:3423. doi: 10.1038/s41467-021-23831-4 (PMC8187657; doi:10.1038/s41467-021-23831-4)
Supplement: Supplementary file 1 — Supplementary Information [file 41467_2021_23831_MOESM1_ESM.pdf]

Supplementary Figures

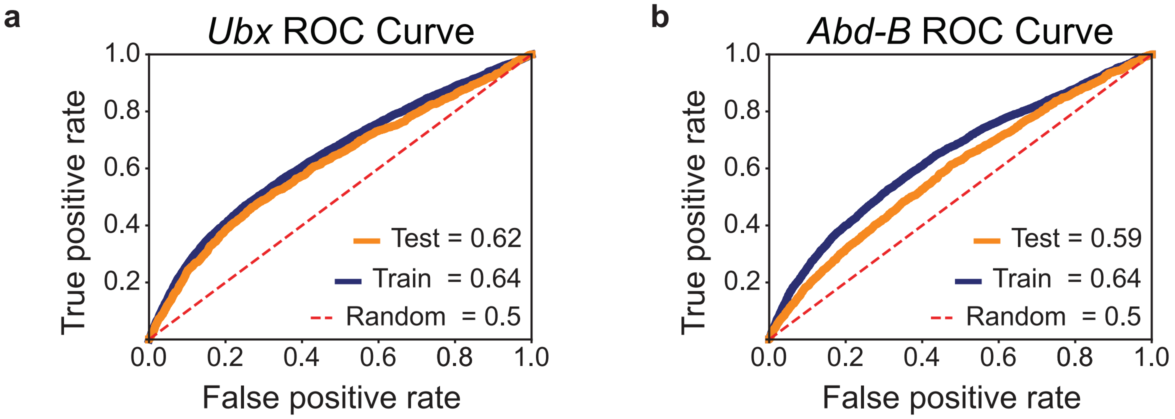

Supp. Fig. 1 | ROC curves for Ubx and Abd-B trained models. **a**, Receiver operating characteristic (ROC) curve of training (blue) and test (orange) datasets for prediction of Ubx expression. Dotted red line represents the performance of random classification. **b**, ROC curve as in (a) for prediction of Abd-B expression.

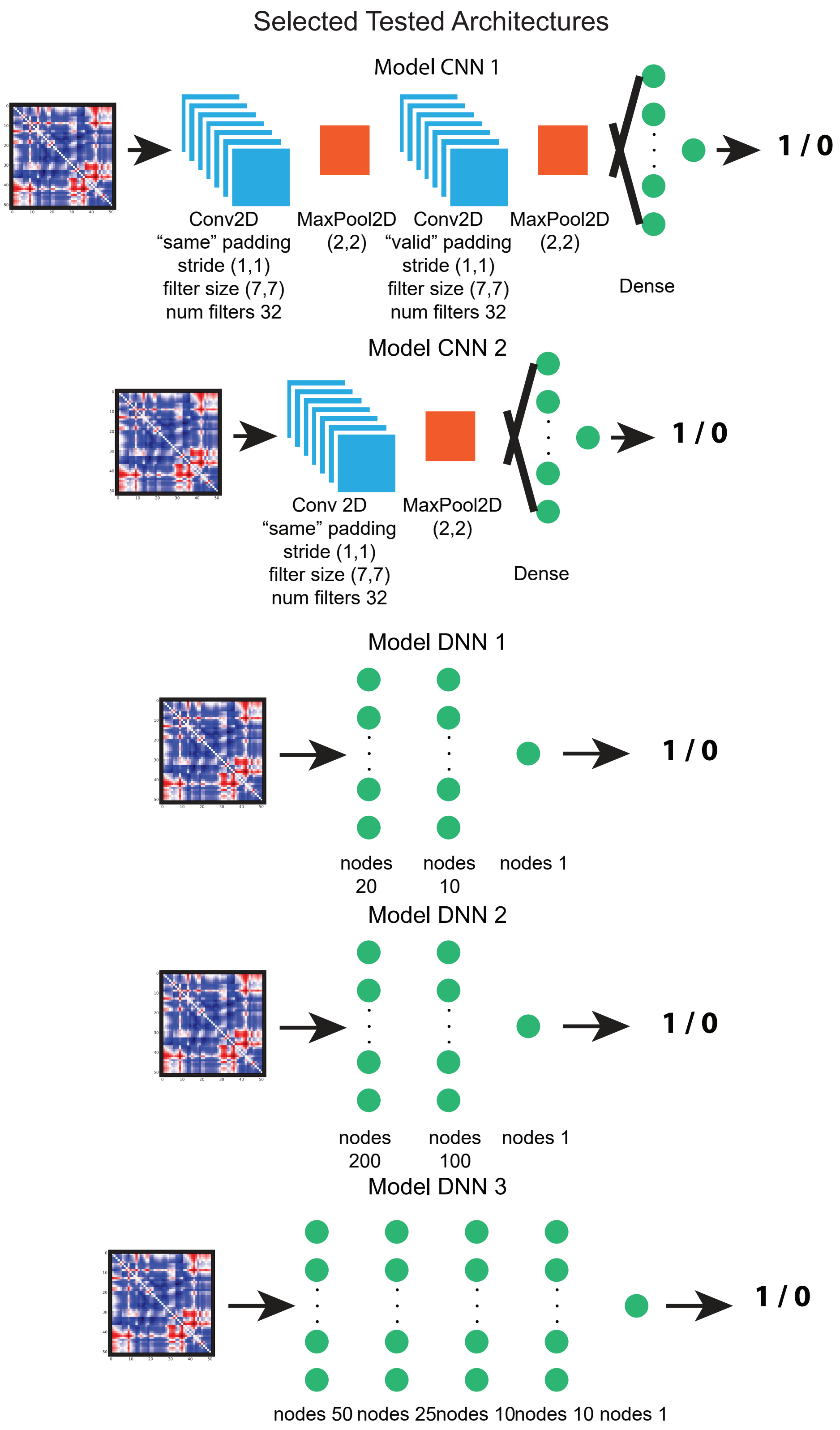

Supp. Fig. 2 | CNN and DNN tested architectures. Schematics of selected Convolutional and Dense Neural Network architectures, corresponding to tested architectures listed in Supplementary Data 3.

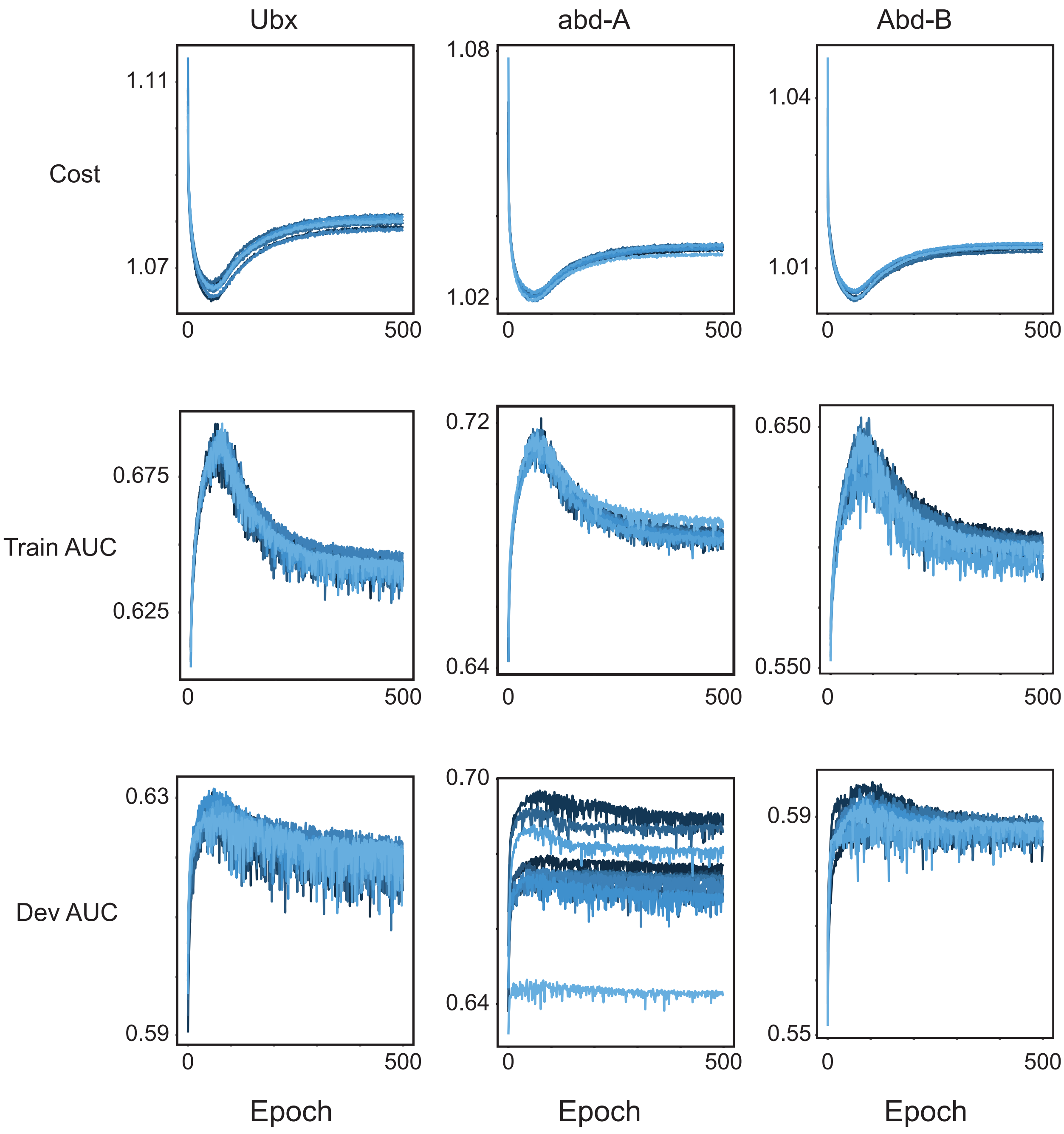

Supp. Fig. 3 | Training cost and score over time. Performance of the CNN is shown across training in terms of cost (weighted cross-entropy loss), training set AUC, and dev/validation set AUC. Metrics are calculated at each epoch. Colored lines indicate different unique cross-validation training runs. See Methods: Deep Learning Models for additional information.

a

Evaluation Metrics for Best Abd-A Model

Dataset	AUC (ROC)	Precision	Recall	F1 Score	Accuracy
Test	0.662246	0.404846	0.357457	0.379679	0.731442
Random Shuffle	0.505994	0.239486	0.211453	0.224598	0.664303

b

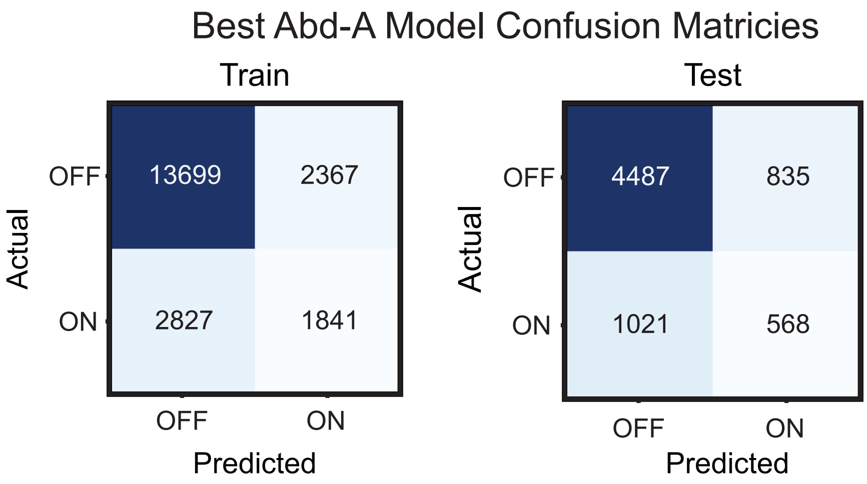

Supp. Fig. 4 | Evaluation Metrics for best abd-A model. a, Results of the best performing abd-A model with multiple metrics, compared to a random shuffle dataset with same proportions as real data. **b,** Confusion matrices showing true positive, true negative, false positive, false negative counts for both train and test sets.

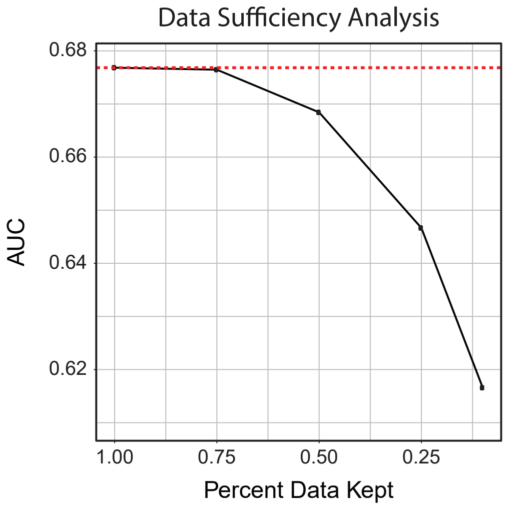

Supp. Fig. 5 | Data Sufficiency Analysis. Results of downsampling number of examples in training dataset to test for data size sufficiency for training CNN model. Y-axis is AUC (ROC) on the validation set after training on a reduced dataset. Red dotted line is the base AUC (ROC) on validation set after training on the full size training dataset. X-axis is the percentage of original number of examples in training data kept.

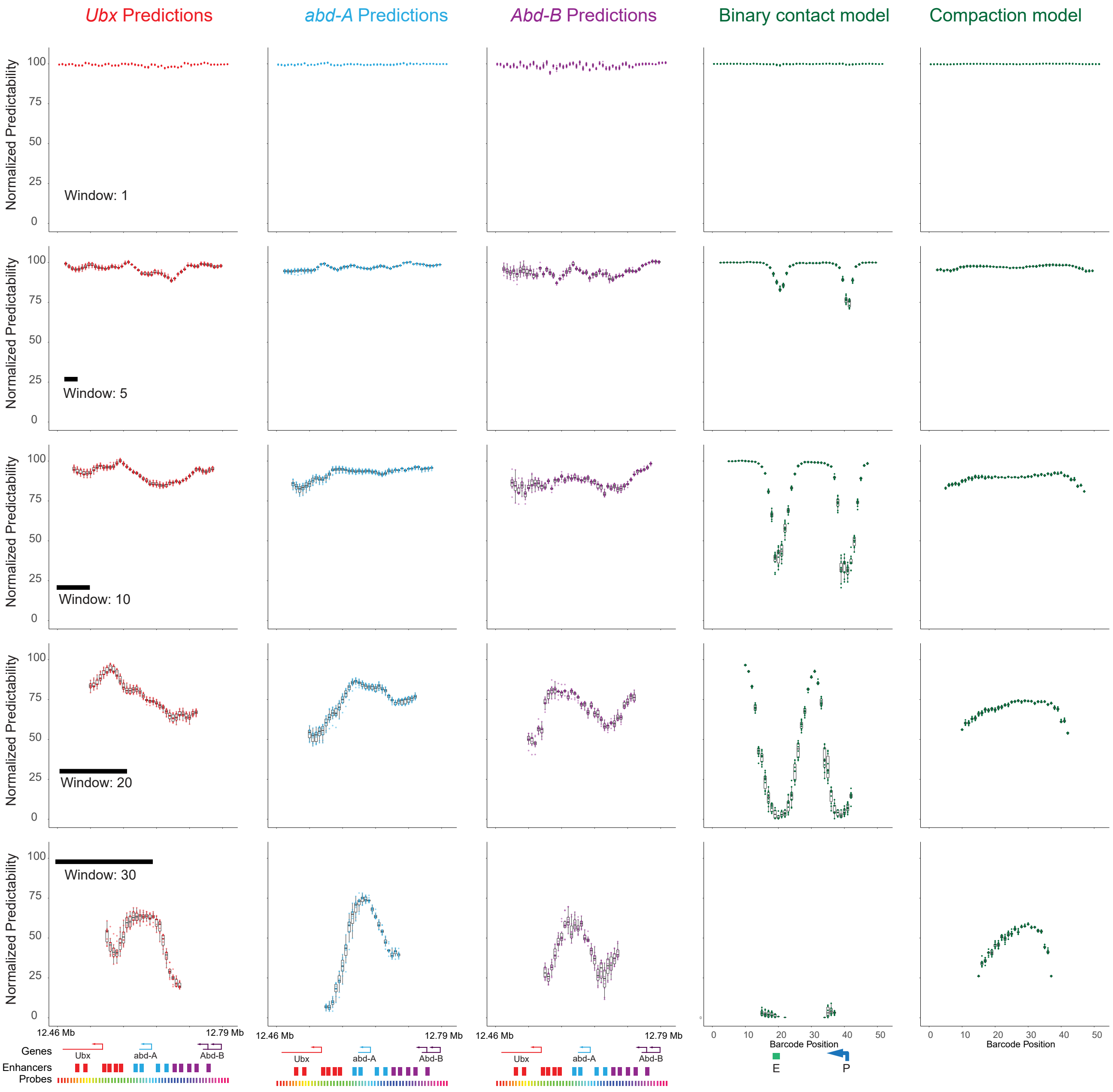

Supp. Fig. 6 | Blanking results for all tested window sizes across E-P contact simulation, compaction simulation, and all three gene CNN models. Rows represent windows of different sizes, from smallest to largest, columns represent different CNN models tested.

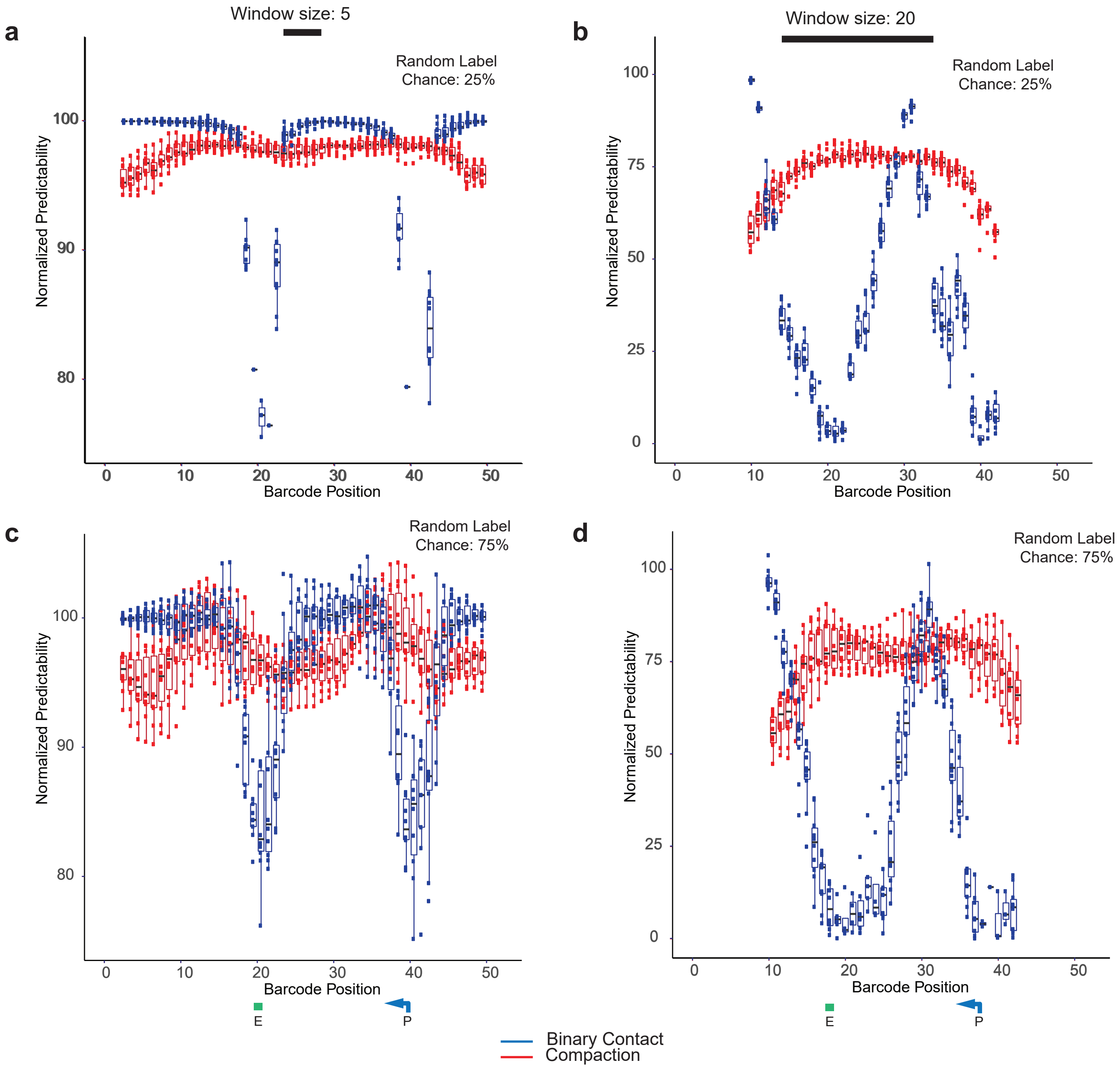

Supp. Fig. 7 | Effect of randomization in the link between the simulated structural rule and transcription state. a, Blanking results for a window size of 5 monomer on two simulations, E-P contact (blue) and compaction (red), both simulations have a 25% chance per example of being assigned a random label instead of being active during E-P contact and silent otherwise. Results show normalized predictability, where 100 corresponds to the AUC (ROC) without any data removal and 0 corresponds to random performance. The position of the enhancer monomer 20, (green) and promoter, monomer 40 (blue) are indicated. **b** As in (a), but with a blanking window of size 20. **c-d** as in (a-b), with both simulations have an 75% chance per example of being assigned a random label. Results from 10 independent trials used in cross-validation are shown as colored dots, overlaid on the boxplots. Boxplots show the quartile values. Whiskers extend to furthest datapoint within 1.5 of the interquartile range.

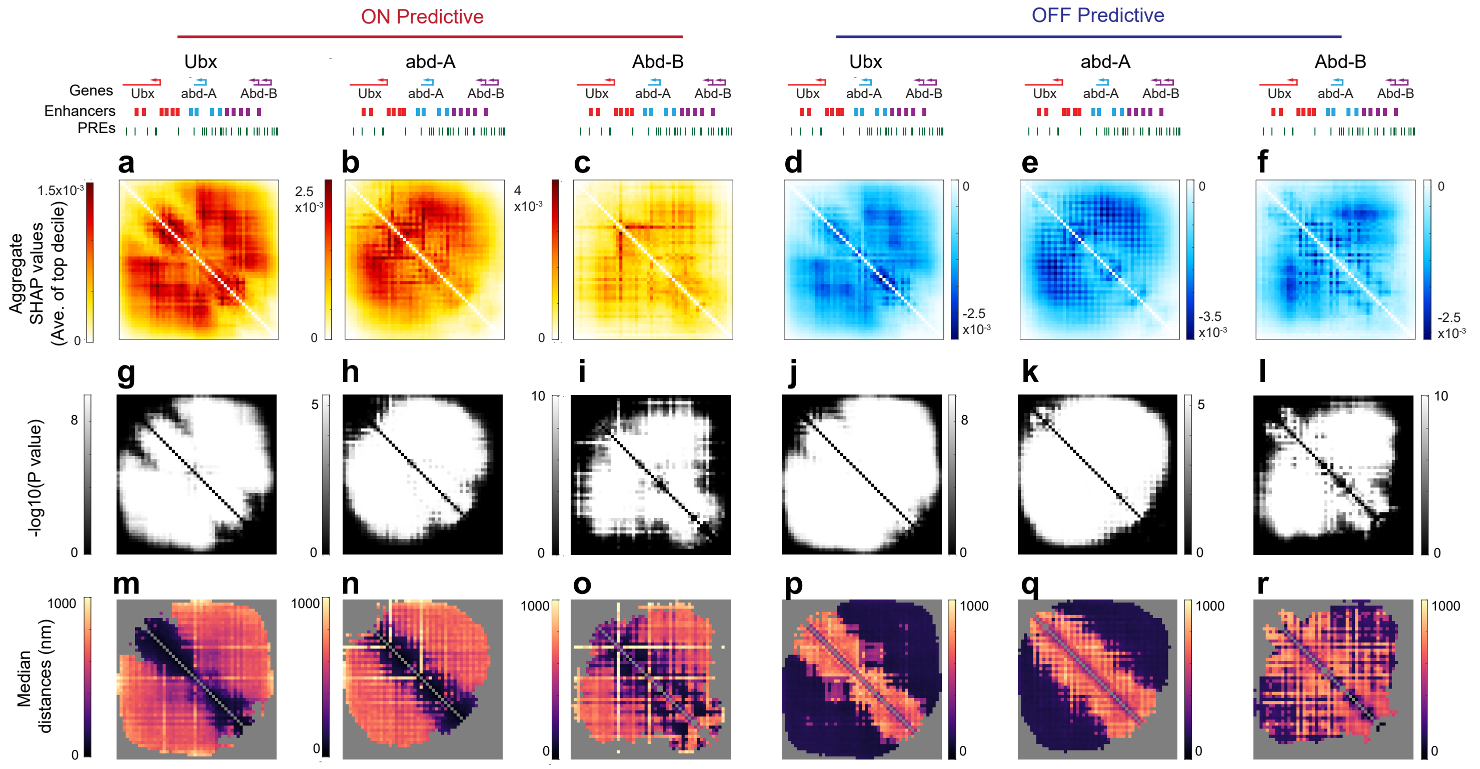

Supp. Fig. 8 | Top 5% Integrated SHAP analysis. Parallel to **Figure 4**, results in terms of top 5% and bottom 5% of SHAP values, instead of top 10% as in Fig. 4. **(a-c)** Top 5% of SHAP values for all pairwise distances from all ON cells for each gene model Ubx, abd-A, Abd-B. **(d-f)** Bottom decile of SHAP values for all pairwise distances from all OFF cells for each gene model, in parallel to a-c. **(g-l)** -log10(P value) maps of SHAP values (a-f). **(m-o)** Median distance among the top decile of SHAP values in a-c. **(p-r)** Median distance among the bottom decile of SHAP values (d-f).

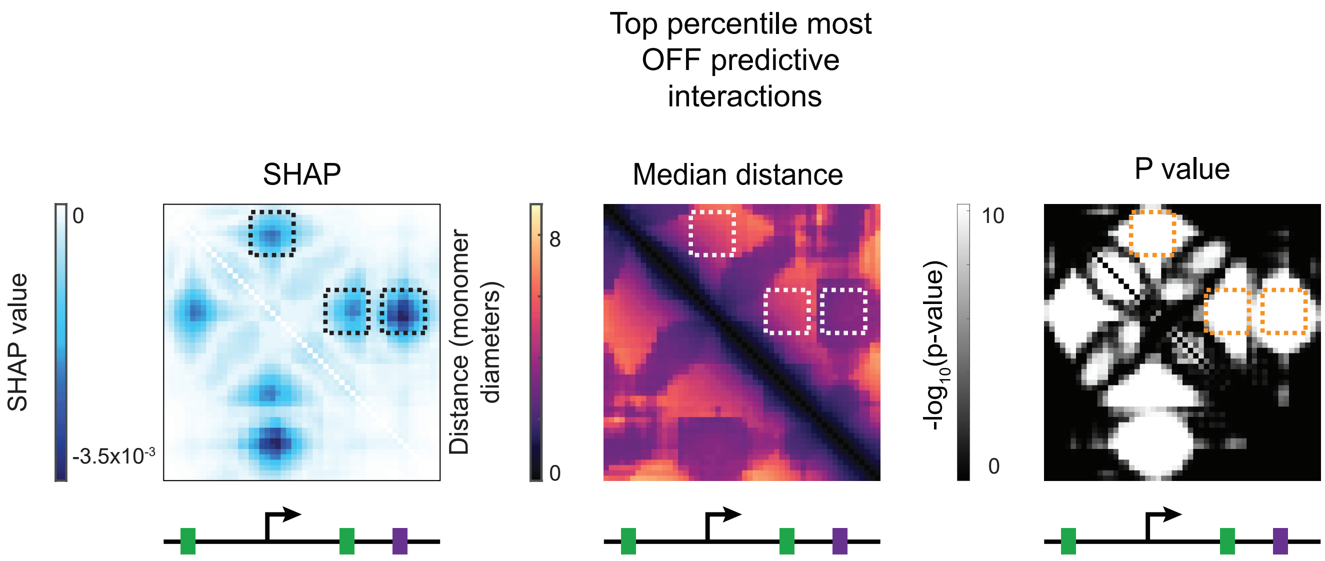

Supp. Fig. 9 | Bottom Decile SHAP analysis of simulation. Population level bottom decile SHAP values for OFF simulation examples, associated median distances, and associated p-values. In parallel to Fig 3g.

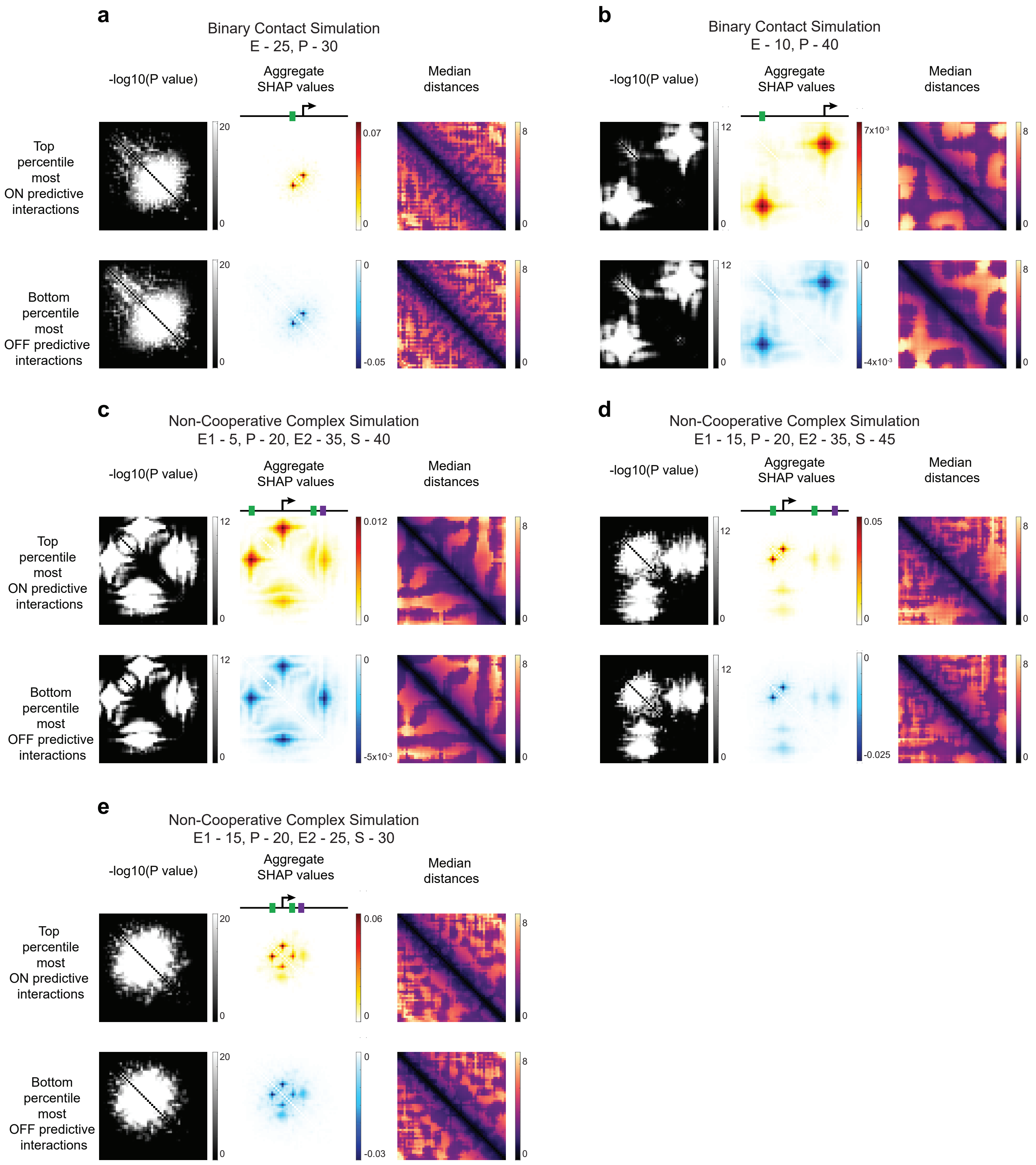

Supp. Fig. 10: E-P Distance variation SHAP analysis. SHAP analysis results for variations of simulated data: binary contact and non-cooperative complex simulation (Fig 3). **(a-b)** SHAP analysis, p-values, and associated median distance maps for ON and OFF cells in binary contact simulation variations: (a) Enhancer at position 25, Promoter at position 30, (b) Enhancer at position 10, Promoter at position 40. **(c-e)** Variations of complex non-cooperative simulation introduced in Fig 3. Enhancers 1,2, Promoter, and Silencer are moved to different positions. SHAP analysis, p values, and associated median distance maps as in a-b.

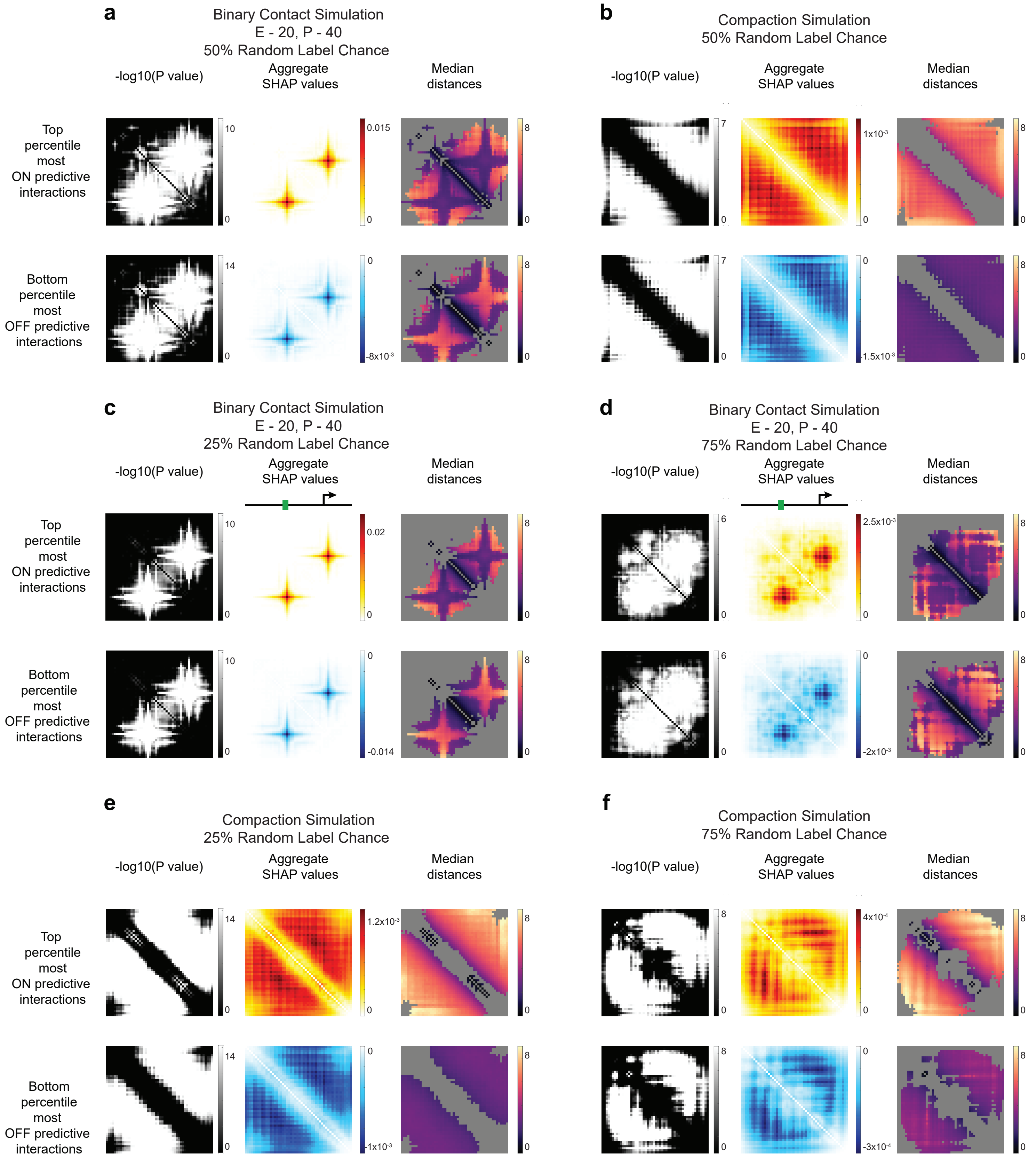

Supp. Fig. 11 | Structure predictivity cutoff variation, SHAP analysis. SHAP analysis results for variations of simulated data: binary contact and compaction simulation (Fig 4). **(a)** SHAP analysis, p-values, and associated median distance maps for ON and OFF cells in binary contact simulation with 50% chance of having transcriptional label ("ON", "OFF") assigned randomly. **(b)** as in (a), but for the compaction simulation. **(c)** as in (a), 25% chance. **(d)** as in (a), 75% chance. **(e)** as in (b), 25% chance. **(f)** as in (b), 75% chance.

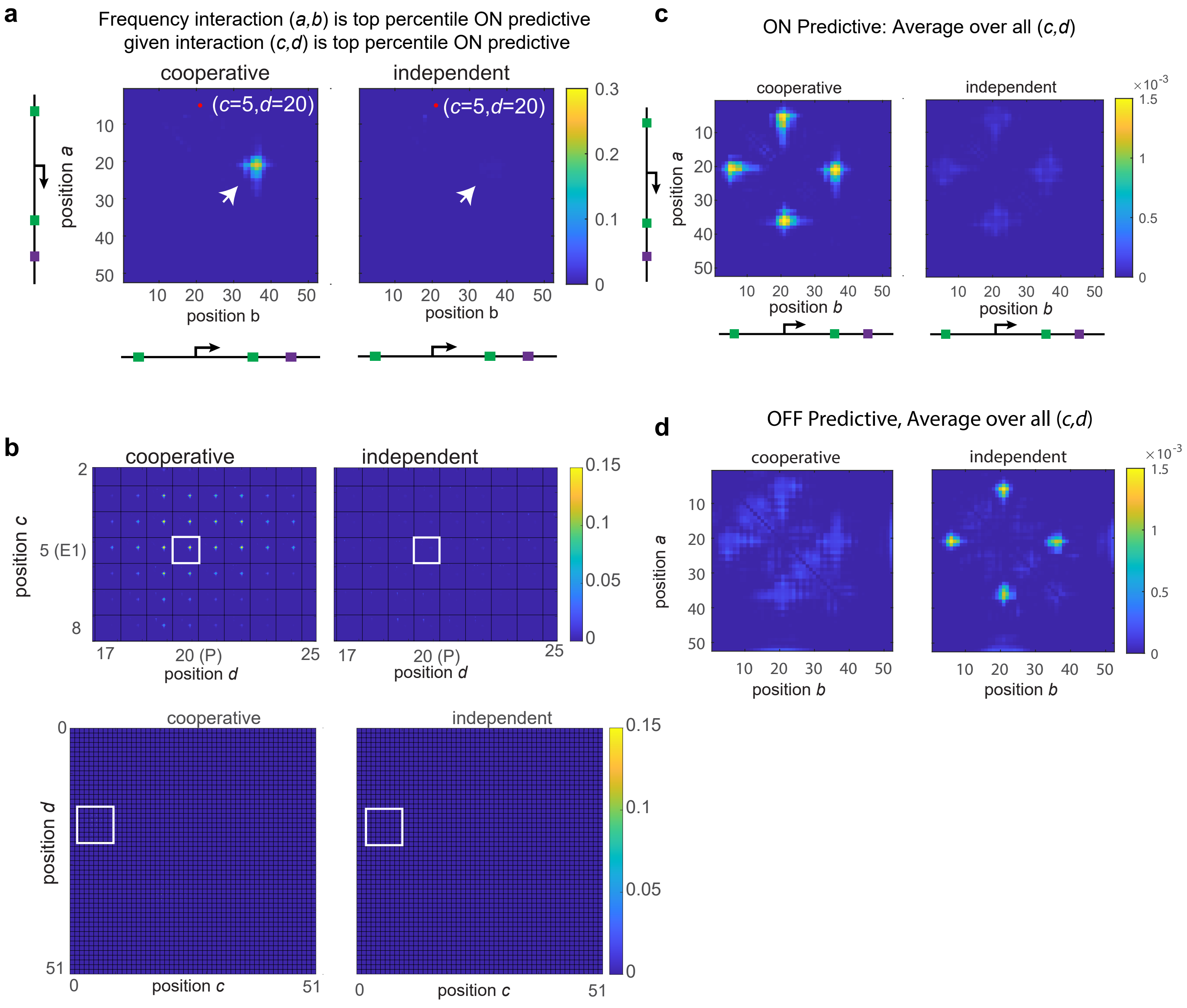

Supp. Fig. 12 | Correlation Analysis Explained. a Example of cooperativity analysis on cooperative and independent simulations with identical positions of elements. For those examples in which the interaction at (5,20) (Enhancer 1, Promoter) has a high SHAP value (in top decile SHAP values at that position), what is the frequency of other interactions in those examples also being in the top decile of SHAP values. At position (20,35) (Promoter, Enhancer 2), a peak of signal is seen in the cooperative simulation only, and none in the independent simulation. **b** Interaction maps computed for all possible combinations of 4 points of interest, Full map and zoom in to interaction at position (5,20) (Enhancer 1, Promoter). **c** Average cooperativity for all interactions for which there exists a cooperative interaction with any other pair, ON cells. **d** as in (c), OFF cells.
