# Supplementary material for: Deep learning connects DNA traces to transcription to reveal predictive features beyond enhancer–promoter contact
Source: Nat Commun. 2021 Jun 8;12:3423. doi: 10.1038/s41467-021-23831-4 (PMC8187657; doi:10.1038/s41467-021-23831-4)
Supplement: Supplementary file 3 — Description of Additional Supplementary Files [file 41467_2021_23831_MOESM3_ESM.pdf]

Description of Additional Supplementary Files

Supplementary Data 1 | Example of DNA trace coordinate data. Single cell example of DNA trace coordinates before removing the rotation component of data. Each trace has 52 individual (x,y,z) coordinates, and associated binary values representing the expression state (1 = ON, 0 = OFF) of each gene (Ubx, abd-A, Abd-B).

Supplementary Data 2 | Cross validation results: CNN. 10-fold stratified cross-validation results for best CNN model for trained models on each of the three genes (*Ubx, abd-A, Abd-B)*. Reported scores are test set AUC (ROC) results.

Supplementary Data 3 | Training parameters and performance.
A grid hyperparameter search was conducted across model architecture (**Supp. Fig. 2**), initial learning rate, weight decay (AdamW optimizer[82]), and minibatch size. Epoch size was held constant at 500 epochs for CNN models, 1000 epochs for DNN models, which were selected well within the training plateau. Training AUC (ROC) and validation/dev set AUC (ROC) were then measured, and the best model selected with the highest Dev Set AUC (ROC). Training was conducted using Tensorflow [79] and Keras [80].

Supplementary Data 4 | Cross validation results: Random Forest. 10-fold stratified cross-validation results for best Random Forest model for trained models on each of the three genes (*Ubx, abd-A, Abd-B)*. Reported scores are test set AUC (ROC) results.
