# Supplementary material for: Deep learning connects DNA traces to transcription to reveal predictive features beyond enhancer–promoter contact
Source: Nat Commun. 2021 Jun 8;12:3423. doi: 10.1038/s41467-021-23831-4 (PMC8187657; doi:10.1038/s41467-021-23831-4)
Supplement: Supplementary file 8 — Reporting Summary [file 41467_2021_23831_MOESM8_ESM.pdf]

nature research

Corresponding author(s): Alistair Boettiger

Last updated by author(s): Dec. 7, 2020

Reporting Summary

Nature Research wishes to improve the reproducibility of the work that we publish. This form provides structure for consistency and transparency in reporting. For further information on Nature Research policies, see Authors & Referees and the Editorial Policy Checklist .

Statistics

For all statistical analyses, confirm that the following items are present in the figure legend, table legend, main text, or Methods section.

n/a	Confirmed	
☐	☒	The exact sample size (*n*) for each experimental group/condition, given as a discrete number and unit of measurement
☐	☒	A statement on whether measurements were taken from distinct samples or whether the same sample was measured repeatedly
☐	☒	The statistical test(s) used AND whether they are one- or two-sided<br>*Only common tests should be described solely by name; describe more complex techniques in the Methods section.*
☐	☒	A description of all covariates tested
☐	☒	A description of any assumptions or corrections, such as tests of normality and adjustment for multiple comparisons
☐	☒	A full description of the statistical parameters including central tendency (e.g. means) or other basic estimates (e.g. regression coefficient) AND variation (e.g. standard deviation) or associated estimates of uncertainty (e.g. confidence intervals)
☐	☒	For null hypothesis testing, the test statistic (e.g. *F*, *t*, *r*) with confidence intervals, effect sizes, degrees of freedom and *P* value noted<br>*Give P values as exact values whenever suitable.*
☒	☐	For Bayesian analysis, information on the choice of priors and Markov chain Monte Carlo settings
☐	☒	For hierarchical and complex designs, identification of the appropriate level for tests and full reporting of outcomes
☐	☒	Estimates of effect sizes (e.g. Cohen's *d*, Pearson's *r*), indicating how they were calculated

Our web collection on statistics for biologists contains articles on many of the points above.

Software and code

Policy information about availability of computer code

Data collection	This computational study relied on data collected in our previous publication, Mateo et al 2019, Nature: (http://dx.doi.org/10.1038/s41586-019-1035-4). Information about data collection can be found in that publication and the corresponding reporting summary.
Data analysis	All data analysis software was custom built for this study, and can be found freely online in an open-source code repository located at: https://github.com/aparna-arr/DeepLearningChromatinStructure. Full descriptions of the methods used can be found in the Methods section. Data analysis was performed primarily in python (v3.8), with some supporting analyses in R and Matlab (R2019a). Polymer simulations were conducted using openmm (v7). Machine learning using Convolutional Neural Networks performed using TensorFlow (v2.2) and Keras (v2.3). Random Forest machine learning was performed using scikit-learn (v0.24).

For manuscripts utilizing custom algorithms or software that are central to the research but not yet described in published literature, software must be made available to editors/reviewers. We strongly encourage code deposition in a community repository (e.g. GitHub). See the Nature Research guidelines for submitting code & software for further information.

Data

Policy information about availability of data

All manuscripts must include a data availability statement. This statement should provide the following information, where applicable:

- Accession codes, unique identifiers, or web links for publicly available datasets
- A list of figures that have associated raw data
- A description of any restrictions on data availability

The data analyzed in this study has been deposited on Zenodo, where it is freely available to the public: https://zenodo.org/record/4741214. DOI: 10.5281/zenodo.4741214

Field-specific reporting

Please select the one below that is the best fit for your research. If you are not sure, read the appropriate sections before making your selection.

☒ Life sciences ☐ Behavioural & social sciences ☐ Ecological, evolutionary & environmental sciences

For a reference copy of the document with all sections, see nature.com/documents/nr-reporting-summary-flat.pdf

Life sciences study design

All studies must disclose on these points even when the disclosure is negative.

Sample size	Sample size was determined from a data sufficiency test, which can be found in Supplementary Materials. This showed that the number of examples in our dataset was sufficient for training and consistent results.
Data exclusions	No data were excluded.
Replication	All results were reproduced as described and indicated in the corresponding data panels. No data failed to reproduce. 10-fold cross validation was used for all neural networks. No experiments were performed in this study. Two biological replicated datasets from the previous study were combined to increase the depth of data available for deep learning.
Randomization	Individual cells were randomly sorted into train/validation/test sets, and 10-fold cross-validation was performed with stratified random shuffling, to preserve class ratios.
Blinding	No blinding was used in these experiments. Blinding was considered unnecessary as the data were randomly sorted into training and validation sets as described above.

Reporting for specific materials, systems and methods

We require information from authors about some types of materials, experimental systems and methods used in many studies. Here, indicate whether each material, system or method listed is relevant to your study. If you are not sure if a list item applies to your research, read the appropriate section before selecting a response.

Materials & experimental systems

n/a	Involved in the study	
☒	☐	Antibodies
☒	☐	Eukaryotic cell lines
☒	☐	Palaeontology
☐	☒	Animals and other organisms
☒	☐	Human research participants
☒	☐	Clinical data

Methods

n/a	Involved in the study	
☒	☐	ChIP-seq
☒	☐	Flow cytometry
☒	☐	MRI-based neuroimaging

Animals and other organisms

Policy information about studies involving animals; ARRIVE guidelines recommended for reporting animal research

Laboratory animals	The data in this study came from wild type embryos (Drosophila melanogaster), both male and female, as described previously, but no new experiments were conducted.
Wild animals	No wild animals were used in these studies.
Field-collected samples	No field collected samples were used in these studies.
Ethics oversight	No ethics approval was required for this study, as no new animal experiments were performed and all data used was from previously published data in Mateo et al. 2019 (Nature).

Note that full information on the approval of the study protocol must also be provided in the manuscript.
